# Progress of Metal Chalcogenides as Catalysts for Efficient Electrosynthesis of Hydrogen Peroxide

**DOI:** 10.3390/ma17174277

**Published:** 2024-08-29

**Authors:** Jeong-Hyun Kim, Jeong-Gyu Lee, Min-Jae Choi

**Affiliations:** 1 Department of Advanced Battery Convergence Engineering, Dongguk University, Seoul 04620, Republic of Korea; jhkim9723@dongguk.edu (J.-H.K.); 2023120388@dgu.ac.kr (J.-G.L.); 2 Department of Chemical & Biochemical Engineering, Dongguk University, Seoul 04620, Republic of Korea

**Keywords:** metal chalcogenides, electrosynthesis, hydrogen peroxide, catalysts

## Abstract

Hydrogen peroxide (H_2_O_2_) is a high-demand chemical, valued as a powerful and eco-friendly oxidant for various industrial applications. The traditional industrial method for producing H_2_O_2_, known as the anthraquinone process, is both costly and environmentally problematic. Electrochemical synthesis, which produces H_2_O_2_ using electricity, offers a sustainable alternative, particularly suited for small-scale, continuous on-site H_2_O_2_ generation due to the portability of electrocatalytic devices. For efficient H_2_O_2_ electrosynthesis, electrocatalysts must exhibit high selectivity, activity, and stability for the two-electron pathway-oxygen reduction reaction (2e^−^ ORR). Transition-metal chalcogenide (TMC)-based materials have emerged as promising candidates for effective 2e^−^ ORR due to their high activity in acidic environments and the abundance of their constituent elements. This review examines the potential of TMC-based catalysts in H_2_O_2_ electrosynthesis, categorizing them into noble-metal and non-noble-metal chalcogenides. It underscores the importance of achieving high selectivity, activity, and stability in 2e^−^ ORR. By reviewing recent advancements and identifying key challenges, this review provides valuable insights into the development of TMC-based electrocatalysts for sustainable H_2_O_2_ production.

## 1. Introduction

Hydrogen peroxide (H_2_O_2_) serves as a potent oxidizing agent, characterized by its odorless, colorless, and slightly acidic properties [1,2,3,4]. Widely utilized across various industrial sectors, including bleaching [4,5], wastewater treatment [6,7], and chemical synthesis [4,8], the H_2_O_2_ market reached approximately USD 3.86 billion in 2023 [9]. It is particularly prevalent in industries such as semiconductor manufacturing, chemical processing, and healthcare [1,2,3]. Its notable environmental advantages over chlorine-based oxidants stem from its decomposition into water and oxygen, producing no harmful byproducts. Additionally, its potential evaluation as an energy carrier, possibly replacing hydrogen in fuel cells for electric vehicles, forecasts a surge in demand [4,10].

To date, around 95% of industrial H_2_O_2_ is produced using the anthraquinone process. However, this method necessitates expensive noble-metal catalysts, primarily palladium, and poses environmental issues due to its toxic byproducts [1,2,4]. Additionally, its continuous operation under high temperature and pressure renders it complex, large-scale, and energy intensive [11]. Another method to directly synthesize H_2_O_2_ is chemically catalyzing a liquid-phase mixture of H_2_ and O_2_ [12]. However, due to the safety hazards associated with H_2_ and O_2_ mixtures, the H_2_ gas must be diluted in CO_2_ or N_2_, significantly limiting production efficiency [3]. Addressing these challenges, the electrosynthesis of H_2_O_2_ has attracted attention due to its green chemistry approach, as it can be conducted under ambient conditions and avoids the use of toxic solvents and intermediates associated with traditional methods [3,13,14]. This process primarily involves the reduction of oxygen in aqueous electrolytes, resulting in minimal byproducts and waste [15]. Furthermore, the electrochemical route can be powered by renewable energy sources, significantly reducing the carbon footprint of H_2_O_2_ production [16]. In addition, the electrochemical process can be integrated with practical fuel cells for renewable energy and simultaneous power generation. Due to the flexibility of the electrochemical system and the portability of electrocatalytic devices, it is also suitable for small-scale, continuous on-site H_2_O_2_ production [13,14,15].

The efficient electrosynthesis of H_2_O_2_ through the oxygen reduction reaction (ORR) requires electrocatalysts with high two-electron pathway (2e^−^) selectivity, activity, and stability, while also being cost-effective for practical applications [3,13,15]. Recently, carbon materials have been extensively studied as potential electrocatalysts, achieving significant advancements in the electrosynthesis of H_2_O_2_ through 2e^−^ ORR due to their high surface area, tunable electronic properties, low conjugated π-system and excellent electrical conductivity [17,18,19,20]. However, most carbon nanomaterials have exhibited high performance in alkaline media and showed poor stability in acidic conditions [21,22,23].

To overcome these limitations, transition-metal chalcogenide (TMC)-based electrocatalysts have emerged for the 2e^−^ ORR owing to their strong stability against acid conditions [23,24,25]. TMCs can be classified into two categories: those composed of noble metals such as palladium (Pd) and platinum (Pt), and those made up of non-noble metals like iron (Fe), cobalt (Co), nickel (Ni), manganese (Mn), and copper (Cu). These metals are further combined with chalcogenides, such as sulfur (S), selenium (Se), and tellurium (Te). Noble-metal chalcogenides (e.g., PdS, PtSe) are known for their high catalytic activity and stability, while their practical applications are often limited by the high cost and scarcity of noble metals [26,27]. In contrast, non-noble-metal chalcogenides (e.g., Cu_2_Se, CoSe_2_, NiTe_2_) offer a cost-effective and abundant alternative, whereas their 2e^−^ ORR activity is still in need to be improved [28,29,30]. 

This review examines the potential of TMC-based catalysts for efficient H_2_O_2_ electrosynthesis via the ORR. First, we will discuss the mechanism of H_2_O_2_ synthesis through 2e^−^ ORR. This includes the competitive reaction, reaction kinetics, and pH-dependent variation in 2e^−^ ORR. Next, recent advancements in TMC electrocatalysts will be covered, which highlight the benefits of phase engineering and the achievements of high performance in terms of selectivity, activity, and stability. This review also covers a comprehensive examination of recent research, including innovative strategies such as chemical transformation and surface coordination tuning, which have shown promising results in various studies. By offering a detailed overview of the current state of research and identifying critical challenges and future directions, this review aims to offer valuable insights into the advancements in TMC-based electrocatalysts for sustainable and efficient H_2_O_2_ production.

## 2. Electrochemical H_2_O_2_ Synthesis through 2e^−^ ORR

### 2.1. Mechanism of Electrocatalytic 2e^−^ ORR

The electrochemical synthesis of H_2_O_2_ primarily involves the ORR. The ORR can proceed via two pathways: the two-electron (2e^−^) pathway producing H_2_O_2_, and the four-electron (4e^−^) pathway producing water (H_2_O) [16]. The 2e^−^ pathway leads to the formation of H_2_O_2_, which is useful in various chemical synthesis and environmental applications. The 4e^−^ pathway is preferred for applications like fuel cells where the complete reduction of oxygen to water is desired, as it provides the highest energy efficiency [31,32]. The desired pathway for H_2_O_2_ production is the 2e^−^ ORR, which can be represented by the following reactions [33]:

In acidic media:

O_2_ + 2H^+^ + 2e^−^ → H_2_O_2_ (E^0^ = 0.70 V vs. SHE) (1)

In alkaline media:

O_2_ + H_2_O + 2e^−^ → HO_2_^−^ + OH^−^ (E^0^ = -0.076 V vs. SHE) (2)

where SHE is a Standard Hydrogen Electrode. However, the 2e^−^ pathway is thermodynamically less favorable compared to the 4e^−^ pathway. The stability of the produced H_2_O_2_ is another challenge, as it can be further reduced to H_2_O or decompose chemically. The 4e^−^ pathway for H_2_O production can be represented by the following reactions [33]:

In acidic media:

O_2_ + 4H^+^ + 4e^−^ → 2H_2_O (E^0^ = 1.23 V vs. SHE) (3)

In alkaline media:

O_2_ + 2H_2_O + 4e^−^ → 4OH^−^ (E^0^ = 0.40 V vs. SHE) (4)

The efficient electrochemical synthesis of H_2_O_2_ hinges on the development of specialized catalysts that can selectively facilitate the 2e^−^ ORR pathway, thereby preventing the further reduction of H_2_O_2_ to H_2_O. This necessitates a focus on materials that not only demonstrate high catalytic performance but also maintain stability under operational conditions.

**Figure 1:**
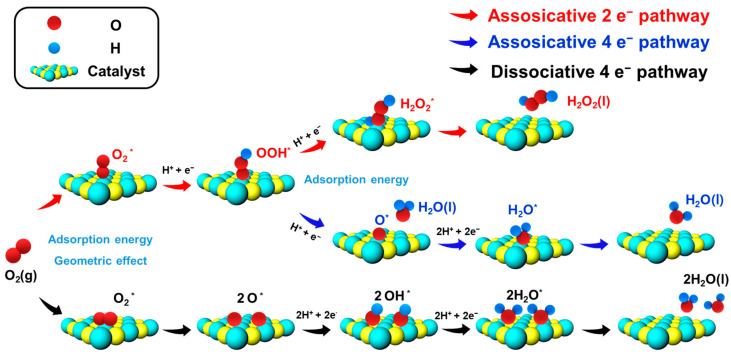
Schematic illustration of associative and dissociative ORR mechanisms. In electrochemical ORR, the 2e^−^ pathway (red) proceeds via the preservation of the O-O bond on the *OOH reaction intermediate. In contrast, the 4e^−^ pathway (blue and black) involves the cleavage of the O-O bond to form *O, ultimately leading to the production of H_2_O.

In catalytic reactions, factors influencing reaction pathways include electrostatic interactions governing catalyst-reactant affinity and steric hindrance dictating reactant adsorption on catalysts [3,34]. The mechanism of electrochemical ORR has been extensively investigated and is depicted in Figure 1. In the ORR, the way oxygen binds to the catalyst, whether in side-on or end-on configurations, dictates pathway preference. The end-on binding of O_2_ triggers the formation of *OOH as an intermediate species (Equation (5)), regardless of the eventual product outcome. If the interaction between *OOH and the catalyst surface remains weak or intermediate, H_2_O_2_ is produced as the ultimate product via the 2e^−^ reaction pathway (Equation (6)). Conversely, if there is a strong binding between *OOH and the catalyst surface, the O-O bond dissociates, resulting in the formation of ^*^O as an intermediate species (Equation (7)). This leads to consecutive 2e^−^ reactions, finally resulting in the 4e^−^ reaction pathway to produce H_2_O as the final product (Equation (8)).

O_2_ + * + (H^+^ + e^−^) → *OOH (5)

*OOH + (H^+^ + e^−^) → H_2_O_2_ + * (6)

*OOH + (H^+^ + e^−^) → *O + H_2_O (7)

*O + (2H^+^ + 2e^−^) → *OH + (H^+^ + e^−^) → H_2_O + * (8)

In the case of a side-on reaction, O_2_ strongly absorbs onto the surface, leading to the dissociation of the O-O bond and subsequent formation of the *O intermediate species (Equation (9)). The *O intermediate then evolves to form *OH (Equation (10)), ultimately resulting in the formation of H_2_O via the 4e^−^ reaction pathway (Equation (11)).

O_2_ + 2* → 2*O (9)

2*O + (2H^+^ + 2e^−^) → 2*OH (10)

2*OH + (2H^+^ + 2e^−^) → 2H_2_O + * (11)

According to this ORR mechanism, adjusting the binding orientation of O_2_ or the affinity between the catalyst and reactants is crucial to produce H_2_O_2._ Effective catalyst design strategies must focus on maintaining the integrity of the O-O bond while also managing factors such as mass transport and electrolyte pH to ensure H_2_O_2_ stability [16]. The adsorption energy refers to the energy change when an O_2_ molecule binds to the surface of the catalyst. It should be sufficiently strong to ensure that O_2_ molecules are effectively adsorbed and activated for the reaction. However, it must not be so strong that it impedes the subsequent desorption of the reaction intermediates of the final products, which would otherwise hinder the overall catalytic efficiency [3,15,32,33]. To achieve optimal selectivity toward H_2_O_2_, catalysts must minimize the kinetic barriers for O_2_ adsorption and *OOH desorption, while maximizing the kinetic barriers for *OOH dissociation to *O and *OH. This balance ensures high reduction activity while maintaining selectivity toward H_2_O_2_. Geometric effects significantly influence the activity and selectivity of the ORR [35,36,37]. These effects pertain to the spatial arrangement and physical structure of the catalyst surface, which play a crucial role in the adsorption and reaction dynamics of O_2_ molecules during the ORR process [38,39]. The atomic arrangement on the catalyst surface, determined by its crystallographic facets, exhibits different structural characteristics [40,41,42]. Catalysts with well-structured surfaces that expose a high number of active sites tend to provide more opportunities for O_2_ adsorption and reaction, thereby demonstrating superior performance [43]. Furthermore, the presence of large groups on the catalyst surface can obstruct O_2_ molecules from accessing the active sites. Such steric hindrance can reduce the number of accessible active sites while also influencing the preferred orientation of the adsorbed O_2_ molecules [44]. To optimize the surface structure, several strategies can be employed, including the creation of porous structures [45,46], introduction of defects [47,48], and construction of heterostructures [49,50]. These methods can adjust the geometric effects and enhance catalytic performance. Moreover, rational reactor and process designs, including conditions of forced convection and low catalyst loading, are crucial for maintaining these conditions.

### 2.2. Reaction Kinetics and Volcano Plot Analysis for 2e^−^ ORR

Based on scaling relationships among various descriptors, specifically ΔG(*OOH) = ΔG(*OH) + 3.2 and ΔG(*O) = 2ΔG(*OH), the adsorption energies of *OH and *OOH serve as crucial indicators for 2e^−^ ORR and 4e^−^ ORR activities [51]. This scaling issue can be mitigated by adjusting the covalency of the catalyst. As electrochemical catalysts have advanced, the complexity of electrode materials has increased, evolving to include doped carbons [52], noble metal alloys [53], and metal oxides [54]. The development of computational methods, such as density functional theory (DFT) calculations, has significantly enhanced the prediction of material activity for H_2_O_2_ electrosynthesis, thereby accelerating catalyst material advancements.

**Figure 2:**
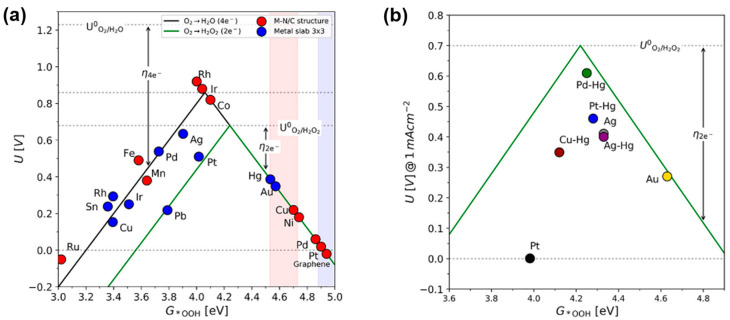
(**a**) Volcano plots illustrating the limiting potential as a function of ΔG*_OOH_ for packed pure metal slabs (blue) and M-N/C structures (red), derived from DFT calculations. The green curve represents the 2e^−^ pathway, while the black curve represents the 4e^−^ pathway. (**b**) Comparison of experimental data and theoretical predictions for pure metals and metal alloys. Circles denote the potential required to achieve a kinetic current density of 1 mA/cm^2^ on polycrystalline electrodes. Reproduced with permission [16]. Copyright 2018, American Chemical Society.

Figure 2a illustrates volcano plots for 2e^−^ ORR and 4e^−^ ORR on metal surfaces and metal–nitrogen/carbon (M-N/C) structures, with dashed lines representing the standard equilibrium potentials of these reactions. The step of O_2_ reduction to *OOH limits the reaction on the weak-binding, right-hand leg of the volcano, while *OOH reduction to H_2_O_2_ limits the reaction on the strong-binding, left-hand leg. Among the pure-metal surfaces, only Au is positioned on the weak-binding leg, whereas among M-N/C structures, Cu-N/C, Ni-N/C, Pd-N/C, and Pt-N/C also lie on the weak-binding leg. Metals with low oxygen affinity maintain low hybridization between metal and oxygen bonds, thus retaining high selectivity for the 2e^−^ ORR [55,56]. Conversely, metals with strong *OH and *OOH adsorption energies promote O-O bond dissociation, limiting the reduction of *OOH to H_2_O_2_ and favoring the 4e^−^ pathway [57]. These differences arise from intrinsic properties such as the number of valence electrons and spin states [58,59]. The optimal catalyst should exhibit moderate *OOH adsorption, balancing the interaction between the catalyst surface and reaction intermediates to achieve excellent performance. The ideal Gibbs adsorption free energy for an H_2_O_2_ synthesis catalyst is approximately 4.22 eV, with a theoretical thermodynamic limit potential of 0.7 V vs. RHE [60]. According to Figure 2a, no material possesses the optimal electronic structure for H_2_O_2_ production. However, alloying Pt and Pd with Hg can adjust the *OOH binding to achieve high activity for H_2_O_2_ production (Figure 2b). Modifying the electronic and geometric effects of catalysts is a valuable approach to controlling catalyst activity and selectivity. Nevertheless, Pt and Pd are rare and expensive metals, imposing significant economic burdens for large-scale industrial applications [61,62]. Hg, while cheaper, is a hazardous metal with considerable environmental and health risks, limiting its industrial use [63]. In contrast, TMCs are significantly cheaper than noble-metal catalysts and are composed of elements abundantly available on Earth, making them economically feasible for large-scale production. Additionally, TMCs exhibit distinctive properties in charge density control, which can be adjusted through various doping and alloying techniques [64]. This capability allows for the development of catalysts with high ORR activity and selectivity. Inconsistencies exist in the kinetic descriptions of different electrode interfaces, such as rotating ring-disk electrodes (RRDEs) and gas-diffusion electrodes (GDEs) [3,14]. In GDEs, the restriction of oxygen diffusion to the reaction site decreases the Faraday efficiency for H_2_O_2_ as catalyst loading increases. High catalyst loading slows the diffusion kinetics of H_2_O_2_, leading to its in situ decomposition within the high-pH electrocatalyst layer [65,66,67]. The mass transport of reaction species or products within the electrode interface microenvironment significantly affects the 2e^−^ ORR kinetics [68]. Electrode structure and interfacial hydrophobicity can also profoundly impact 2e^−^ ORR kinetics [69]. Constructing three-phase sites, tailoring local solution environments, and designing flow-through reactors can facilitate O_2_ uptake and proton transfer, thereby enhancing the 2e^−^ ORR kinetics [3,34,70].

### 2.3. Effects of Electrolyte pH on ORR Mechanism

The pH of the electrolyte significantly impacts the activity and selectivity of catalysts used in the ORR. This is because the behavior of the reaction pathway, including multiple electron transfer steps and the polarity of solvated water, varies under different pH conditions [71,72,73]. Understanding these effects is crucial for designing and optimizing efficient electrocatalysts.

**Figure 3:**
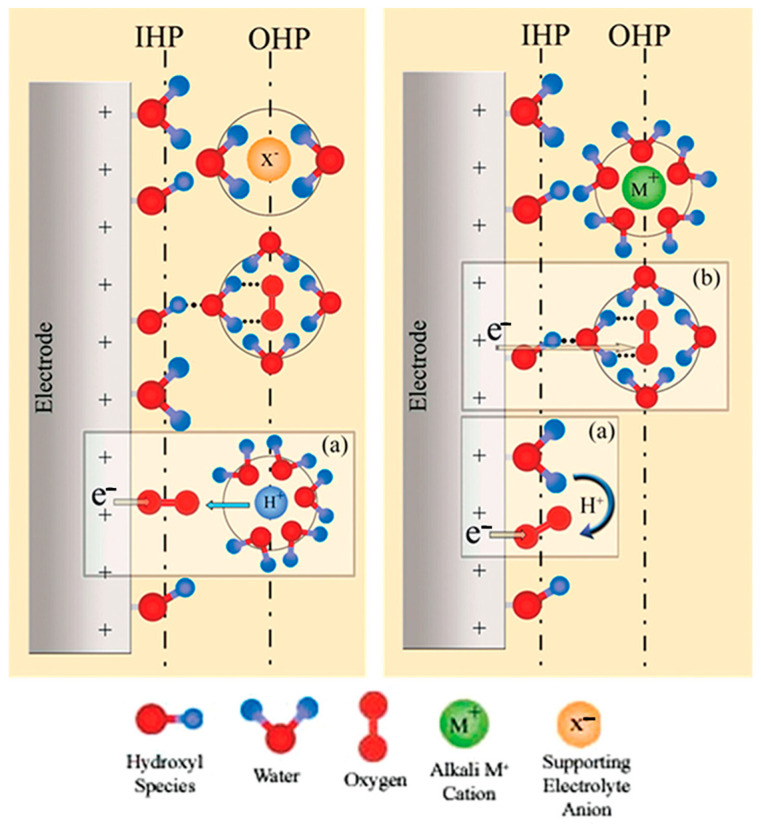
Schematic depiction of the double-layer structure during ORR in acidic (**left**) and alkaline (**right**) environments. Insets (**a**) and (**b**) illustrate the inner-sphere and outer-sphere electron transfer processes, respectively. The inner Helmholtz plane (IHP) and the outer Helmholtz plane (OHP) are shown, highlighting the distribution of hydroxyl species, water, oxygen alkali metal cations, and supporting electrolyte anions. Reproduced with permission. Copyright 2011 [74], American Chemical Society.

Figure 3 illustrates the double-layer structure scenario at the electrode/electrolyte interface during ORR. The interface between the electrode and electrolyte forms a double-layer structure, comprising the inner Helmholtz plane (IHP) and the outer Helmholtz plane (OHP). The IHP is populated by specifically adsorbed hydroxyl species, solvent water dipoles oriented with the oxygen towards the electrode surface, and chemisorbed O_2_. Beyond the OHP, charged species are loosely scattered in the aqueous solution [74]. The species occupying the IHP and OHP vary under different pH conditions, and their interactions promote either an inner-sphere electron transfer (ISET) process or an outer-sphere electron transfer (OSET) process [4,75].

In acidic environments, the ORR generally follows an ISET mechanism. In this mechanism, molecular O_2_ strongly chemisorbs onto the catalyst surface, proceeding through a series of reduction steps. The 4e^−^ pathway is predominant in acidic media and involves the following reaction steps [22]:

O_2_ + 4H^+^ + 4e^−^ → 2H_2_O (12)

The process includes intermediates such as *O_2_, *OOH, *O, and *OH, which remain adsorbed on the active site until the final product, H_2_O, is desorbed into the bulk electrolyte. The specific steps are as follows:

O_2_ → *O_2_ (13)

*O_2_ + H_2_O + 2e^−^ → *OOH^−^ + OH^−^ (14)

*OOH^−^ + H_2_O + 2e^−^ → 3OH^−^ (15)

All intermediates stay bound to the catalyst surface, ensuring efficient electron transfer and product formation. In alkaline media, the ORR often follows an OSET mechanism. Here, solvated molecular oxygen (O_2_·(H_2_O)_n_)_aq_ interacts with surface hydroxyl species (*OH) via hydrogen bonding. This promotes the formation of superoxide and hydroperoxide intermediates, which are crucial for the 2e^−^ pathway. For the 2e^−^ pathway specific to alkaline conditions, the reactions are as follows [4]:

M-OH + [O_2_·(H_2_O)_n_]_aq_ + e^−^ → M-OH + *OOH^−^ + OH^−^ + (H_2_O)_n−1_ (16)

*OOH^−^ + e^−^ → *OOH^−^ (17)

*OOH^−^ → (OOH^−^)_aq_ (18)

In this reaction, the superoxide ion (*OOH^−^) acts as a major intermediate, easily converting to H_2_O_2_. The weak hydrogen bonds between solvated molecular oxygen and surface hydroxyl species facilitate the outer-sphere electron transfer, promoting the formation of H_2_O_2_. The bond dissociation energy (BDE) of O_2_ is 498 kJ/mol, larger than the BDE of many typical nonmetal–O bonds. Thus, the dissociation of O_2_ is more energetically favorable on a catalyst surface with moderate adsorption energy of O_2_ through various intermediates such as adsorbed oxygen (O_ad_), superoxide ion (O_2_^−^), and HO_2,ad_, among others [33].

The pH conditions in which the ORR occurs play a crucial role in determining the reaction pathways and the stability of the reaction products. This understanding is vital for developing catalysts optimized for specific pH conditions, particularly in electrochemical H_2_O_2_ production. In alkaline media, ORR predominantly follows the 2e^−^ pathway, leading to the production of H_2_O_2_ [4,76]. However, a significant challenge associated with this pathway is the inherent instability of H_2_O_2_ in alkaline environments, where it is prone to further reduction or decomposition into H_2_O. This instability can severely limit the efficiency of H_2_O_2_ production. To address this, strategies such as rational reactor design or the introduction of H_2_O_2_ stabilizers are being explored to enhance the stability and yield of H_2_O_2_ in alkaline conditions [4]. Conversely, in acidic conditions, ORR tends to follow the 4e^−^ pathway, which results in the complete reduction of O_2_ to H_2_O. While the 2e^−^ pathway that leads to H_2_O_2_ formation is less active in acidic environments, the H_2_O_2_ that is produced is generally more stable and less prone to decomposition [77]. The stability offers a distinct advantage in applications where the production and accumulation of H_2_O_2_ are desired.

The decomposition of H_2_O_2_ varies significantly between acidic and alkaline conditions. In acidic media, the following reaction occurs [33]:

H_2_O_2_ + 2H^+^+ 2e^−^ → 2H_2_O (E^0^ = 1.776 V vs. SHE) (19)

In alkaline media, the decomposition proceeds via the following:

HO_2_^−^ + H_2_O + 2e^−^ → 3OH^−^ (E^0^ = 0.878 V vs. SHE) (20)

These reactions illustrate the different pathways through which H_2_O_2_ can be decomposed depending on the pH, underscoring the importance of developing catalysts that possess high stability and performance in acidic environments to effectively inhibit these decomposition reactions. Such advancements could significantly improve the efficiency of H_2_O_2_ production, with wide-ranging implications for its use in chemical synthesis and environmental applications.

## 3. Metal Chalcogenide Catalysts for H_2_O_2_ Electrosynthesis

Materials known for 2e^−^ ORR electrocatalysis to date include carbon-based materials [26,78,79,80,81,82,83], noble metals and their alloys [55,56,84,85], and single-atom-based materials [86,87,88,89]. However, due to several limitations, applying these materials to industrial applications remains challenging. Carbon-based materials such as O-CNTs have exhibited high selectivity for the 2e^−^ ORR in alkaline electrolytes [90,91,92]. Nevertheless, these materials generally demonstrate low activity in acidic electrolytes, which poses a significant drawback [91,92,93].

The utilization of carbon materials that exhibit low current densities in acidic media presents significant limitations. In the electrosynthesis of H_2_O_2_, the choice of electrolyte is a crucial factor. In alkaline electrolytes, H_2_O_2_ decomposes rapidly, necessitating immediate use or the addition of stabilizers such as phosphoric acid for long-term use [4]. Conversely, the electrosynthesis of H_2_O_2_ in acidic electrolytes offers greater stability by preventing the decomposition of H_2_O_2_ [94]. This eliminates the need for stabilizers, thereby reducing production costs and making it immediately applicable to various processes such as the Electro-Fenton method [25,95]. In acidic media, Pt- and Pd-based alloys and single-atom catalysts exhibit significant 2e^−^ ORR activity and selectivity [84,85,93]. However, Pt- and Pd-based alloys are hindered by issues of scarcity and high costs. Single-atom catalysts (SACs) are prone to agglomeration, leading to catalyst deactivation [51]. Additionally, the preparation of efficient SACs involves complex processes, including high-temperature annealing and acid treatment [96,97,98,99]. Therefore, extensive research is being conducted to discover alternative electrocatalysts that are earth-abundant, cost-effective, and exhibit excellent activity and stability in acidic electrolytes.

TMCs have garnered significant research interest over the past few decades due to their earth abundance, versatile redox properties, and facile synthesis, making them suitable for large-scale applications. Numerous studies have extensively investigated TMCs as active catalysts for electrocatalytic reactions such as the oxygen evolution reaction (OER), hydrogen evolution reaction (HER) [100,101], and 4e^−^ ORR [64], demonstrating high activity and significant potential in electrosynthesis [102]. However, investigations on 2e^−^ ORR catalysts are still in their early stages. Several studies have highlighted that TMCs, such as CoS_2_, CoSe_2_, MoTe_2_, and NiS_2_ [103,104,105,106,107], demonstrate promising selectivity and activity in acidic environments, emphasizing their potential as 2e^−^ ORR catalysts. These materials not only exhibit excellent selectivity and activity but also possess remarkable stability in acidic conditions. The ability of TMCs to exhibit these characteristics can be attributed to their unique structural properties, such as high designability of morphology, electronic structures, and rich active sites [108,109]. In the 2e^−^ ORR mechanism, achieving the appropriate adsorption energy for the reaction intermediate *OOH on the active sites is crucial [10,110]. TMCs can provide diverse and specific surface structural motifs with controllable tunability, enabling the rational design of TMCs with optimal *OOH adsorption energy. Such a design would prevent O-O bond cleavage and facilitate the desorption of hydrogen peroxide during the reduction process. Therefore, further research on TMCs for the 2e^−^ ORR is urgent.

In this chapter, we classify ORR catalysts into noble-metal chalcogenides and non-noble-metal chalcogenides and present the reaction mechanisms for the 2e^−^ ORR pathway in both acidic and alkaline environments. We also discuss the key factors that enhance selectivity and provide theoretical analyses.

### 3.1. Material Design for Efficient Electrocatalysts

As illustrated in Table 1, the activity and selectivity of TMC-based 2e^−^ ORR electrocatalysts have been greatly enhanced due to the implementation of diverse material design approaches. The pure TMCs are generally inefficient as electrocatalysts for H_2_O_2_ production. To improve their performance, strategies such as alloying, element doping, defect engineering, and facet engineering have been employed to modulate the electronic structure, thereby adjusting the binding energy of the *OOH intermediate, which prevents O-O bond cleavage and enhances selectivity.

The electronic structure plays a crucial role in determining the performance of electrocatalysts and can be modified through strategies like doping, defect engineering, and other approaches. Altering the electronic structure leads to the redistribution of spin and charge, which in turn enhances electrocatalytic performance [115]. The presence of defects in heterogeneous catalysts can disrupt the original stoichiometric ratio, cause distortions in the electric field, and alter the electronic distribution. These changes have been extensively studied to adjust intrinsic physicochemical properties and improve electrocatalytic performance. By carefully creating defects of various types, quantities, and locations, it is possible to precisely control the resulting electrocatalytic performance [116].

Guo et al. synthesized PtCu catalysts with Cu-vacancy defects through an electrochemical etching process. These PtCu catalysts, which have an ultrafine size and self-supporting rigid structure, exhibited significant structural stability. Density functional theory (DFT) calculations showed that the introduction of Cu vacancies on the surface of Pt-Cu (111) catalysts enhances the adsorption capacity of Pt atoms for the *OH intermediate while simultaneously weakening the adsorption for the *O intermediate. This catalyst demonstrated a mass activity 14.1 times higher than that of the commercial Pt/C catalyst (20 wt%, JM) [117]. Wang et al. synthesized selenium-vacancy-enriched Fe-CoSe-HT catalysts through a straightforward hydrothermal process followed by high-temperature annealing. The doping of trace amounts of Fe was found to influence crystal growth and induce the formation of Se vacancies during the annealing process. When applied to the 2e^−^ ORR, the Fe-CoSe-HT catalyst achieved a H_2_O_2_ production rate of 18.37 mmol L^−1^ over 120 min under neutral conditions, with a Faraday efficiency of 83.2%. The selenium vacancies in Fe-CoSe-HT significantly improved the selectivity of the 2e^−^ ORR and accelerated Fe reduction, leading to enhanced ∙OH radical generation and the effective treatment of organic pollutants [118].

### 3.2. Noble-Metal Chalcogenides

Noble-metal catalysts are widely used in many electrochemical catalysis fields due to their high stability and excellent performance [119,120,121]. However, they are well known for favoring the 4e^−^ pathway in ORR reactions, resulting in low selectivity for the electrochemical synthesis of hydrogen peroxide [122,123]. Therefore, research is being conducted to develop catalysts that preferentially facilitate the 2e^−^ pathway.

**Figure 4:**
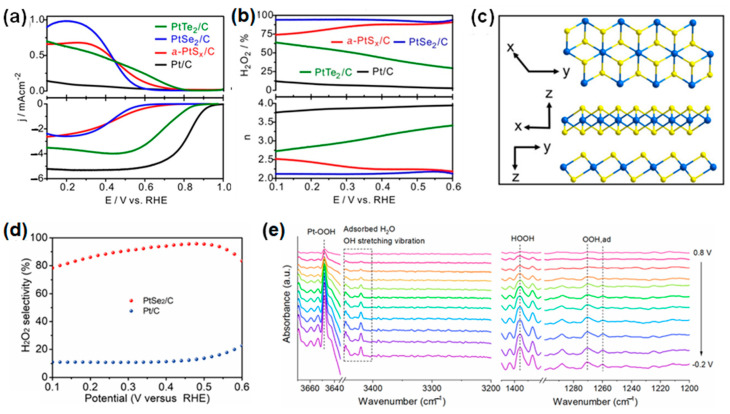
Platinum chalcogenide-based catalysts for 2e^−^ ORR: (**a**) ORR polarization curves and (**b**) corresponding *n* and H_2_O_2_ selectivity of Pt/C, a-PtS*_x_*/C, PtSe_2_/C, and PtTe_2_/C in 0.1 M HClO_4_. Reproduced with permission [27]. Copyright 2023, American Chemical Society. (**c**) Crystal structure of PtSe_2_ nanosheets. (**d**) Selectivity of PtSe_2_/C and Pt/C at 1600 rpm in O_2_ saturated 1 M PBS electrolytes. (**e**) In situ ATR-IR spectra under applied potentials of PtSe_2_/C (OOH, ad: adsorbed OOH). Reproduced with permission [26]. Copyright 2022, The Royal Society of Chemistry.

To induce the 2e^−^ pathway in ORR, noble metal alloys such as Pt-Hg and Pd-Hg are widely used [55,85]. However, Hg is highly toxic and easily leaches out during the ORR reaction, making practical applications challenging. Compared to metal alloys, noble metal-based chalcogenides with high electronegativity can easily modulate the electronic structure and exhibit high catalytic activity, showing great potential for future applications in hydrogen peroxide production. Song et al. [27] introduced individual chalcogens into platinum metal and compared the catalytic activity for the 2e^−^ pathway based on the atomic radius and chemical properties of the chalcogen elements, confirming the order PtTe_2_/C < a-PtS_2_/C < PtSe_2_/C (Figure 4a). Among these, PtSe_2_/C exhibited high selectivity (~91%) and high mass activity (439 A/g_Pt_) for the 2e⁻ pathway (Figure 4b). They effectively modulated the surface atomic arrangement and electronic structure of the active sites using chalcogens. Platinum chalcogenides with isolated active sites facilitated end-on adsorption of O_2_, and by adjusting the binding energy of *OOH through changes in electronic structure, PtSe_2_/C favored the 2e^−^ pathway.

Utilizing the advantages of metal selenides, Dong et al. [26] successfully synthesized ultrathin single-crystal PtSe_2_ nanosheets through a selenization process using commercial Pt/C as a precursor. As shown in Figure 4c, these PtSe_2_ nanosheets exhibit an interlayer atomic arrangement of Se-Pt-Se within a hexagonal crystal structure. Unlike Pt/C, which follows a 4-electron pathway, PtSe_2_ demonstrates an exclusive 2e^−^ pathway, showing high selectivity for H_2_O_2_ and high current density across a wide pH range (up to 94.1%) (Figure 4d). In situ attenuated total reflection Fourier transform infrared (ATR-FTIR) spectroscopy confirmed the catalytic active center of Pt, with the adsorption of the reaction intermediate *OOH and the formation of HOOH clearly observed (Figure 4e). Despite this, many studies have been conducted on the use of platinum chalcogenide electrocatalysts as cathode catalysts in fuel cells, favoring the 4e^−^ reaction [124,125]. Therefore, further research in this area will be necessary.

### 3.3. Non-Noble-Metal Chalcogenides

Noble metal compounds are known for their high selectivity and stability for H_2_O_2_ production even at small overpotentials. However, their high cost poses challenges for practical industrial applications. In contrast, non-noble transition-metal chalcogenides have surface structures with controllable tunability and are abundant and easily obtainable, making them promising catalysts for efficient H_2_O_2_ production [126]. Recent studies have confirmed that non-noble transition-metal chalcogenide catalysts exhibit high 2e^−^ ORR activity. By comparing their selectivity of 2e^−^ ORR, stability, and yields, future research directions for practical applications can be better envisioned.

#### 3.3.1. Cobalt Chalcogenides

Among the transition metals (Mn, Fe, Co, Ni, Cu), cobalt, with its optimal d band center, is favorable for the 2e^−^ reaction because its binding strength with the intermediate (*OOH) is neither too strong nor too weak [93]. Consequently, cobalt has been extensively studied as a 2e^−^ ORR catalyst.

**Figure 5:**
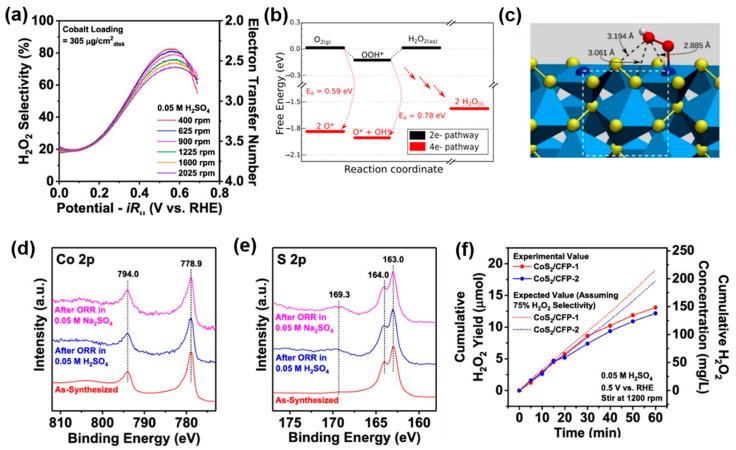
(**a**) H_2_O_2_ selectivity in O_2_-saturated 0.05 M H_2_SO_4_ of drop-casted CoS_2_ (cobalt loading = 305 μg/cm_disk_^2^) at different rotation rates. (**b**) Computational modeling of ORR on the CoS_2_ (100) surface. Free-energy diagram for both 2e^−^ and 4e^−^ ORR at the calculated standard equilibrium reduction potential of 2e^−^ ORR. (**c**) Side view of the CoS_2_ surface with adsorbed *OOH. (**d**) Co 2p and (**e**) S 2p XPS spectra (structural and compositional characterizations of drop-casted CoS_2_ before and after operational stability tests in O_2_-saturated 0.05 M H_2_SO_4_ and 0.05 M Na_2_SO_4_). (**f**) Electrocatalytic production of H_2_O_2_ on CoS_2_/CFP in a three-electrode H-cell setup, and chemical quantification of the H_2_O_2_ product at 0.5 V vs. RHE in O_2_ saturated 0.05 M H_2_SO_4_ and the corresponding cumulative H_2_O_2_ yield. Reproduced with permission [103]. Copyright 2019, American Chemical Society.

Sheng et al. [103] demonstrated through computational and experimental studies that cobalt pyrite (CoS_2_) nanomaterials, which are abundant on Earth, are highly active and selective for the 2e^−^ ORR. CoS_2_ nanomaterials drop-casted on a RRDE exhibited a high onset potential and selectivity (0.69 V vs. RHE onset potential, ~70% selectivity) in 0.05 M H_2_SO₄ electrolytes (Figure 5a). DFT modeling revealed that the surface energy of the CoS_2_ (100) facet was lower than that of the (110) or (111) facets, indicating higher stability. Additionally, on the (100) facet, the binding of *OOH at the Co active sites occurred more readily, resulting in an energy barrier of 0.59 eV, which was lower than the binuclear dissociation barrier between neighboring Co sites (0.78 eV) (Figure 5b). This structural characteristic of CoS_2_ ensures optimal binding of *OOH without dissociation of the O-O bond, making CoS_2_ favorable for hydrogen peroxide production. Furthermore, while most metal surfaces interact with the *OOH intermediate in a side-on manner, the isolated active sites on the CoS_2_ surface allow for the end-on binding of ^*^OOH (Figure 5c). X-ray Photoelectron Spectroscopy (XPS) analysis conducted before and after operational stability tests in both acidic and neutral electrolytes confirmed that the binding energies of the predominant Co 2p (778.9 and 794.0 eV) and S 2p (163.0 and 164.0 eV) signals matched the literature values (Figure 5d,e). This consistency indicates that the surface chemical states remained unchanged, demonstrating excellent stability. Additionally, CoS_2_ nanowires directly grown on high-surface-area carbon fiber paper demonstrated high hydrogen peroxide accumulation rates and excellent durability in a three-electrode H-cell setup (Figure 5f).

**Figure 6:**
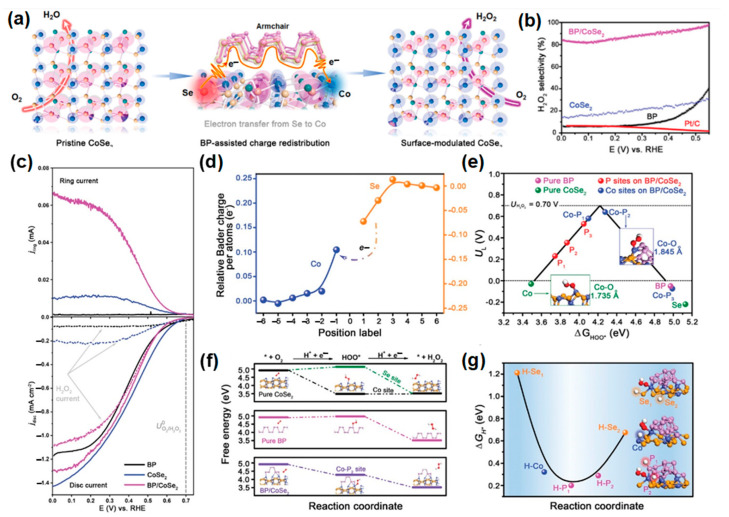
(**a**) Schematic of the BP-mediated surface charge redistribution of CoSe_2_ for enhanced 2e^−^ ORR process. (**b**) Comparison of ORR performance (up) and the simultaneous H_2_O_2_ detection current densities at the ring electrode (down) for pure CoSe_2_, BP, and BP/CoSe_2_ catalysts in O_2_-saturated 0.5 m H_2_SO_4_ at a sweep rate of 10 mV s^−1^. (**c**) H_2_O_2_ selectivity as a function of the applied potential of different catalysts. (**d**) Bader charge depth profile of Co and Se atoms for the BP/CoSe_2_ catalyst. The relative Bader charge represents the difference between the average Bader charge of Co/Se atoms in each layer of BP/CoSe_2_ and CoSe_2._ (**e**) Calculated 2e^−^ (solid line) volcano plot for the electrochemical ORR to H_2_O_2_ demonstrated with the limiting potential plotted as a function of ΔG*_OOH_ (the equilibrium potential: 0.70 V). (**f**) Free-energy diagrams of 2e^−^ ORR on pure CoSe_2_, pure BP, and BP/CoSe_2_ catalysts in acidic electrolyte at zero potential. (**g**) Hydrogen adsorption free energy for the protonation of *OOH to H_2_O_2_ at different sites of the BP/CoSe_2_ catalyst. Reproduced with permission [105]. Copyright 2022, Wiley-VCH GmbH.

As noted in many papers on fuel cell catalysts, metal chalcogenide materials do not always induce a 2e^−^ ORR. However, by adding new materials or modifying the surface to redistribute electron density, it is possible to increase the selectivity for the 2e^−^ reaction in catalysts that are otherwise favorable for the 4e^−^ reaction. Zheng et al. [105] demonstrated this through the synthesis of BP/CoSe_2_, where the low selectivity of CoSe_2_ nanobelts for hydrogen peroxide was improved by functionalizing them with few-layer black phosphorus (BP) nanosheets, enabling surface charge redistribution in CoSe_2_ (Figure 6a). As a result, the presence of BP increased the selectivity for hydrogen peroxide production from 30% to 90% (Figure 6b,c). In the DFT calculations, Bader charge depth analysis showed that Co atoms on the BP/CoSe_2_ surface have increased electron density, while Se atoms exhibit decreased charge density (Figure 6d). These computational results suggest that BP induces charge redistribution on the CoSe_2_ surface by facilitating electron transfer from Se to Co atoms. In Figure 6e, the coupling of BP with CoSe_2_ optimizes the ΔG*_OOH_ value to 4.28 eV at the Co-P_2_ site, facilitating surface charge redistribution. Additionally, it shows an increase in the Co-O bond length from 1.74 Å to 1.85 Å, enhancing the efficiency of hydrogen peroxide production. As shown in Figure 6f, the coupling of BP with CoSe_2_ optimizes *OOH adsorption at the Co-P_2_ site, improving the 2e^−^ ORR pathway and avoiding O-O bond cleavage. Furthermore, BP/CoSe_2_ enhances H_2_O_2_ production efficiency by facilitating *OOH hydrogenation at P sites with lower free energy compared to Se sites (Figure 6g). Additionally, when quantifying H_2_O_2_ formation every hour at a constant current density of 54 mA/cm^2^ in a flow reactor, we confirmed that the BP/CoSe_2_ catalyst could maintain approximately 90% H_2_O_2_ selectivity over a 5 h period. Therefore, optimizing the atomic structure and electronic configuration confirms that the efficient electrochemical synthesis of hydrogen peroxide can be achieved.

#### 3.3.2. Nickel Chalcogenides

Nickel is an earth-abundant element known for its high activity and strong resistance to corrosion, making it an attractive energy electrocatalyst. Among transition metals, nickel has a preferential adsorption capacity for oxygen molecules [23,127]. Additionally, nickel chalcogenide catalysts have been reported to exhibit high stability and good efficiency in strongly acidic electrolytes without undergoing corrosion.

**Figure 7:**
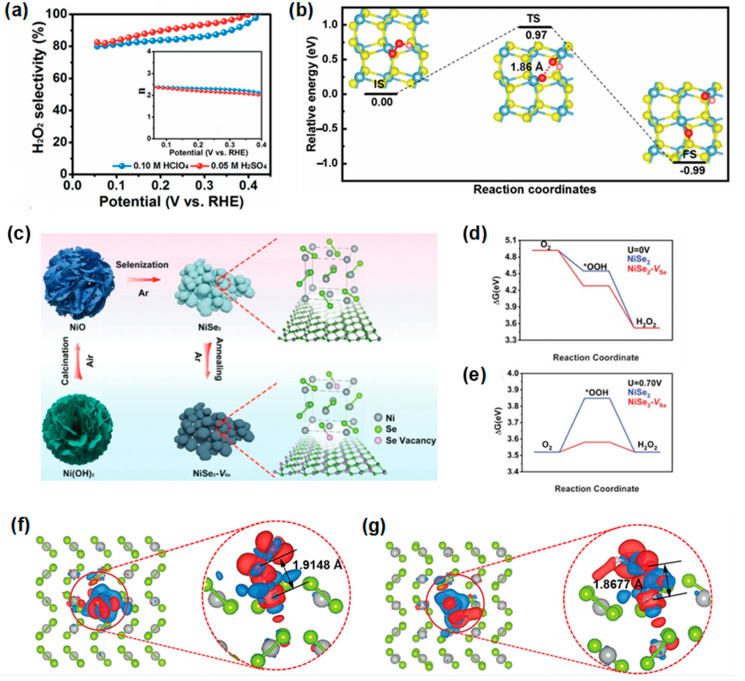
(**a**) Calculated H_2_O_2_ selectivity and the electron transfer numbers (inset in (**a**)) of NiS_2_ based on the RRDE measurements in O_2_-saturated 0.05 M H_2_SO_4_ electrolytes and 0.10 M HClO_4_, respectively. (**b**) The minimum-energy pathway for the dissociation of *OOH to *O + *OH. The blue, yellow, red, and pink spheres denote the Ni, S, O, and H atoms, respectively. Reproduced with permission [107]. Copyright 2021, The Royal Society of Chemistry. (**c**) The synthetic scheme of NiSe_2_-V_Se_ nanoparticles. Free-energy diagram for 2e^−^ ORR on NiSe_2_ and NiSe_2_-V_Se_ at (**d**) U = 0 V and (**e**) U = 0.70 V. Differential charge density distribution between adsorbed *OOH and (**f**) NiSe_2_ and (**g**) NiSe_2_-V_Se_ substrates, where the grey and green spheres represent the Ni and Se atom, respectively, while the red- and blue-colored isosurfaces mean the positive and negative charge, respectively. Reproduced with permission [111]. Copyright 2022, Wiley-VCH GmbH.

Liang et al. [107] successfully synthesized a NiS_2_ catalyst with high selectivity for the 2e^−^ ORR in acidic electrolytes (up to 99% H_2_O_2_ selectivity) from a hydroxide precursor through a low-temperature sulfurization reaction strategy (Figure 7a). Figure 7b shows that *OOH binds to a single oxygen atom on the surface of NiS_2_, with an energy barrier of 0.97 eV for dissociation, indicating high selectivity towards H_2_O_2_ production by preventing O-O bond cleavage. To further enhance the catalytic performance of the 2e^−^ ORR, defect engineering has been employed to induce charge-transfer and energy-band structure changes, thereby improving catalyst performance in several studies (e.g., Fe_2_O_3_, Ni_2−x_P) [128,129]. In metal chalcogenide catalysts, to increase catalytic selectivity in alkaline electrolytes, Wang et al. [111] successfully synthesized nickel diselenide (NiSe_2_-V_Se_) with intentionally charge-polarized anion vacancies through a sequential solvothermal calcination–selenization–annealing process (Figure 7c). Figure 7d,e show that NiSe_2_-V_Se_ has ΔG*_OOH_ values closer to the ideal ones (4.28 and 3.58 eV at 0 V and 0.70 V, respectively) compared to NiSe_2_ (4.548 and 3.848 eV), indicating enhanced catalytic performance for the 2e^−^ ORR. In Figure 7f,g, the differential charge density distributions between adsorbed *OOH and the surfaces of NiSe_2_ and NiSe_2_-V_Se_ indicate stronger binding in NiSe_2_-V_Se_, with shorter bond lengths of 1.8677 Å compared to 1.9148 Å in NiSe_2_. The combined analysis of DFT calculations confirmed that the presence of charge-polarized Se vacancies influenced the optimization of free adsorption energy of reaction intermediates while reducing electron transfer rates. Also, it demonstrated impressive stability, exhibiting only a minor reduction in selectivity after undergoing 5000 cycles of accelerated degradation testing (ADT). Similarly, Sun et al. [108] compared the catalytic performance of nickel chalcogenides with different anion species (Se^2−^, S^2−^, O^2−^). Like cobalt chalcogenides, this study confirms that the *d*-band center of metal selenides at −2.24 eV is higher, leading to better electrocatalytic performance.

#### 3.3.3. Copper Chalcogenides

**Figure 8:**
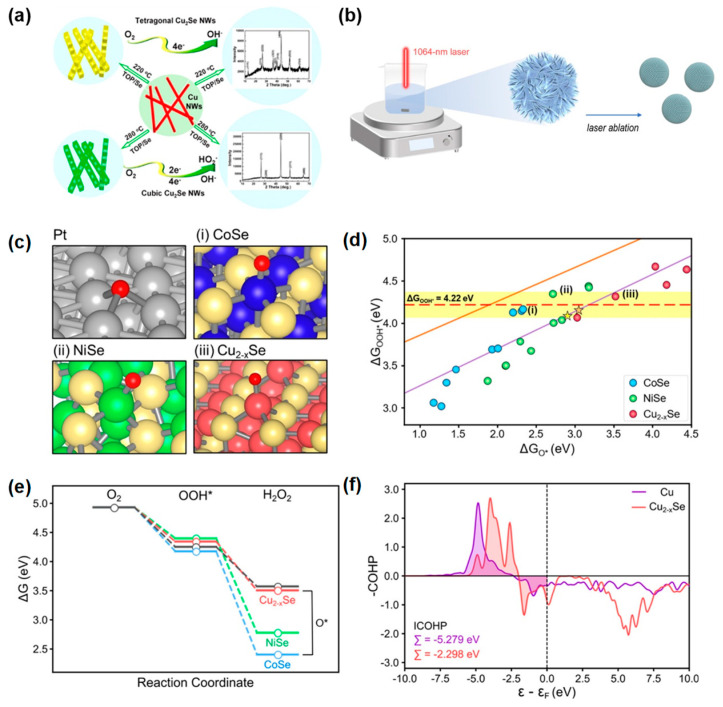
(**a**) The synthesis of pure tetragonal-phase Cu_2_Se NWs and pure cubic-phase Cu_2_Se NWs. Reproduced with permission [30]. Copyright 2013, American Chemical Society. (**b**) The advanced laser ablation method to synthesize M-Se nano-alloys (**c**) Atomic structures of *O adsorption for FCC Pt, (**i**) CoSe, (**ii**) NiSe, and (**iii**) Cu_2-x_Se. (**d**) ΔG_OOH_* and ΔG_O_* of CoSe, NiSe, and Cu_2-x_Se. (**e**) Free-energy diagram of 2e^−^ ORR− at U = 0 V (vs. RHE). Black lines indicate free energies of the ideal 2e^−^ ORR catalyst with η_ORR_ = 0 V. Weaker ΔG_O_* implies higher selectivity for 2e^−^ ORR over 4e^−^ ORR. (**f**) COHP analysis and the corresponding ICOHP for *O adsorption on Cu (111) and Cu_2–x_Se (111). Upper and lower peaks indicate bonding and antibonding states, respectively. Reproduced with permission [112]. Copyright 2021, American Chemical Society.

Cu-based nanostructures have been proposed as ORR catalysts in alkaline electrolytes, showing promise for electrochemical catalytic activity. Liu et al. [30] demonstrated that the rich phase structure of Cu_2_Se allows for the tuning of 2e^−^ or 4e^−^ ORR activity (Figure 8a). Pure tetragonal and cubic Cu_2_Se nanowires (NWs) were obtained at 220 and 280 °C, respectively, by using trioctylphosphine (TOP) as a surfactant to accelerate the transformation reaction. The tetragonal Cu_2_Se NWs follow the 4e^−^ ORR mechanism, while the cubic phase exhibits a dual-path mechanism that includes both 2e^−^ and 4e^−^ pathways. This indicates the potential of copper chalcogenide catalysts for the 2e^−^ pathway. The high selectivity for hydrogen peroxide production in copper selenide was also confirmed in the paper by Yuan et al. [112]. They synthesized a series of nanosized M–Se catalysts (M = Cu, Ni, Co) using a laser ablation method and achieved flexible control over the 2e^−^ or 4e^−^ ORR reactions by engineering the alloying components (Figure 8b). In Figure 8c, these metal chalcogenides (CoSe, NiSe, Cu_7.2_Se_4_) are visualized, showing that surface metal atoms are surrounded by Se atoms, thereby eliminating hollow sites for O adsorption. This arrangement leads to the weaker binding of intermediates, enhancing hydrogen peroxide production. As seen in Figure 8d, the scaling lines for ΔG*_OOH_ and ΔG*_O_ for all three metal selenides shift toward weaker *O binding, significantly improving H_2_O_2_ selectivity compared to pure metals. Among them, Cu_2−_*_x_*Se exhibits the weakest *O binding, showing the highest selectivity for the 2e^−^ reaction (Figure 8e). A crystal orbital Hamiltonian population analysis (COHP) comparing the effects of electronic structures revealed that the bonding state energy of O–Cu_2−_*_x_*Se (111) is higher than that of O–Cu (111), and the filling of antibonding states is more significant, resulting in weaker O–Cu_2−_*_x_*Se (111) bonding. This observation aligns with the experimental data for the 2 e^−^ reaction in Cu_2−_*_x_*Se (Figure 8f).

#### 3.3.4. Other Metal Chalcogenides

Other non-precious-metal chalcogenide compounds, such as molybdenum (Mo) [113]- and zinc (Zn) [113,114]-based chalcogenides, have also been used in ORR.

**Figure 9:**
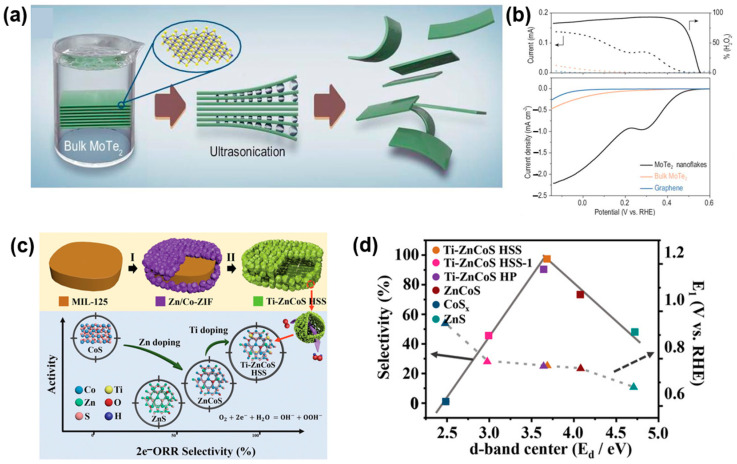
(**a**) Schematic exfoliation process of bulk MoTe_2_ powders to nanoflakes by LPE. (**b**) (**Lower panel**) Polarization curves of MoTe_2_ nanoflakes, bulk MoTe_2_ powders, and graphene nanosheets alone, respectively, and (**upper panel**) the corresponding ring currents (dashed line) and H_2_O_2_ percentage (solid line). Reproduced with permission [113]. Copyright 2024, China Science Publishing & Media Ltd. (Science Press). (**c**) A scheme of the two-step synthesis of Ti–ZnCoS HSS. And illustration of the 2e^−^ ORR activity–selectivity of CoS_x_, ZnS, ZnCoS, and Ti–ZnCoS HSS. (**d**) Relationship between selectivity at 0.55 V (vs. RHE) and activity (potential at 1 mA cm^–2^) and *d*-band center for different catalysts. Reproduced with permission [114]. Copyright 2022, Wiley-VCH GmbH.

Zhao et al. demonstrated that 2H-phase MoTe_2_ nanoflakes are highly efficient 2e^−^ ORR electrocatalysts, achieving a selectivity of around 93% [113]. These MoTe_2_ nanoflakes were synthesized using a top-down liquid-phase exfoliation (LPE) method, where the bulk material was exfoliated into a few stabilized layers during ultrasonic treatment, disrupting the Van der Waals interactions, as shown in Figure 9a. The exfoliated MoTe_2_ nanoflakes showed excellent performance, with an onset potential of approximately 0.56 V in a 0.5 M sulfuric acid electrolyte (Figure 9b). DFT calculations further support that the 2e^−^ ORR is promoted by the favorable binding energy of the *OOH intermediate at the zigzag edge of 2H-MoTe_2_. Zhang et al. synthesized a titanium-doped zinc–cobalt sulfide hollow superstructure (Ti–ZnCoS HSS) that demonstrated high selectivity (98%) and strong activity (1 mA cm^−2^ at 0.774 V) for H_2_O_2_ production in alkaline electrolytes [114]. Figure 9c shows Ti–ZnCoS HSS was created through a two-step synthesis process using NH_2_-MIL-125@ZnCo-ZIF (Zeolitic Imidazolate Framework) as the precursor. Titanium-doping facilitated the formation of a hollow ZnCoS nanocage superstructure. This design optimizes the d-band center and the adsorption energy of intermediates (3.68 eV), resulting in numerous active sites and enhanced 2e^−^ ORR performance (Figure 9d). In addition to the proposed TMCs, there have been many studies on the ORR performance of various non-precious-metal chalcogenide compounds such as MnSe [130], Co_1–x_Zn_x_Se@NCF [131], and GaSe [132]. However, most of these studies have focused on the electrochemical properties of the 4e^−^ pathway rather than the 2e^−^ pathway. Therefore, further research on the electrochemical properties of the 2e^−^ ORR pathway in these promising TMCs is highly anticipated.

## 4. Conclusions

In this review, we have comprehensively explored the recent advancements in the field of TMCs as electrocatalysts for the ORR. Our discussion delved into the intricate ORR mechanisms, the significant impact of pH on the reaction pathways, and the comparative performance of TMC-based catalysts. The insights gathered from this review highlight the promising potential of TMCs in achieving efficient and selective 2e^−^ ORR, a critical step for sustainable H_2_O_2_ production. The ORR mechanism can proceed via the 2e^−^ or 4e^−^ pathway, significantly influenced by the pH of the electrolyte. In acidic media, the ISET mechanism is predominant, whereas in alkaline conditions, the OSET mechanism becomes more significant. Understanding these pathways is crucial for designing effective catalysts. The stability of H_2_O_2_ in acidic conditions compared to its rapid decomposition in alkaline media underscores the importance of selecting appropriate electrolytes to enhance ORR efficiency and selectivity. TMCs, including compounds of cobalt, nickel, and copper, have shown remarkable potential due to their tunable surface structures and electronic properties. These materials provide abundant active sites and exhibit high selectivity and stability in both acidic and alkaline environments. Advanced techniques like phase engineering, defect creation, and heterostructure construction have been employed to optimize the ORR performance of TMCs, demonstrating significant improvements in catalytic activity and selectivity. Despite the progress made, several challenges and opportunities remain in the research and development of TMC-based electrocatalysts for ORR.

(1) Development of high-performing electrocatalysts: TMCs have demonstrated superior catalytic efficiency in acidic media, outperforming carbon-based materials, largely due to their electronic structure. Key strategies such as doping and defect engineering, which modulate electron density, are essential for further performance enhancement. Among TMCs, cobalt chalcogenides have garnered significant interest, owing to the favorable adsorption energies associated with cobalt’s electron density. Future research should prioritize the exploration of charge redistribution on cobalt chalcogenides’ surfaces, employing advanced strategies to drive further performance improvements.
(2) H_2_O_2_ stability and catalyst durability: Hydrogen peroxide is slightly acidic and highly stable in such conditions, allowing it to be maintained without the need for stabilizers. TMCs like BP/CoSe_2_ and NiS_2_ show excellent selectivity and activity in acidic electrolytes, making them strong candidates for 2e^−^ ORR electrocatalysts in practical applications. However, the acidic environment also brings heightened risks of corrosion and degradation, which must be addressed. To ensure these catalysts perform reliably in real-world conditions, future research should focus on improving their long-term stability and durability, such as through the development of carbon shells.
(3) Mechanistic insights: In-depth mechanistic studies using advanced characterization techniques like in situ ATR-IR, in situ Raman, XANES, and EXAFS are necessary to gain a better understanding of the ORR processes at the atomic level. Such insights will aid in the rational design of more effective catalysts.
(4) Scale-up and operational costs: To transition from laboratory-scale experiments to practical large-scale applications, several key challenges must be addressed. These challenges include optimizing reactor design, increasing current density, and ensuring high Faradaic efficiency during prolonged electrolysis. For practical implementation, maintaining high Faradaic efficiency at current densities above 300 mA cm^−2^ is essential. Additionally, reducing stabilizer use in acidic electrolytes, lowering production costs by using affordable non-precious-metal catalysts, and innovating electrochemical reactor designs—such as gas diffusion electrodes (GDEs) and membrane electrode assemblies (MEAs)—are critical for scaling up the ORR process.

In conclusion, the continued development and optimization of TMC-based electrocatalysts hold great promise for advancing sustainable and efficient H_2_O_2_ production through ORR. By addressing the aforementioned challenges and leveraging the unique properties of TMCs, we can pave the way for their practical application in various electrochemical technologies.

## Figures and Tables

**Table 1:** Summary of the TMC-based catalysts for enhanced 2e^−^ ORR electrocatalysis.

Classification	Catalyst	Synthesis Method	Electrolyte	Onset Potential (0.1 mA cm^−1^) [V vs.RHE]	Selectivity [%]	Reference
Noble-metal chalcogenides	PtSe_2_/C	Chemical Vapor Deposition	0.1 M HClO_4_	0.6	~94	[27]
	PtSe_2_/C	Chemical Vapor Deposition	0.1 M HClO_4_	0.7	~94	[26]
Non-noble-metal chalcogenides	CoS_2_	Hydrothermal	0.05 M H_2_SO_4_	0.69	~70	[103]
	BP/CoSe_2_	Hydrothermal	0.5 M H_2_SO_4_	0.68	~90	[105]
	NiS_2_	Hydrothermal–Chemical Vapor Deposition	0.05 M H_2_SO_4_	0.56	~99	[107]
	NiSe_2_-V_se_	Hydrothermal–Calcination-Annealing	0.1 M KOH	0.6	~96	[111]
	Cu_7.2_Se_4_	Chemical Vapor Deposition	0.1 M KOH	0.64	~94	[112]
	2H-MoTe_2_	Liquid Phase Exfoliation	0.5 M H_2_SO_4_	0.56	~93	[113]
	Ti-ZnCoS HSS	Hydrothermal	0.1 M KOH	0.78	~98	[114]

## Data Availability

No new data were created or analyzed in this study.
